# Pathways Connecting Late-Life Depression and Dementia

**DOI:** 10.3389/fphar.2020.00279

**Published:** 2020-03-13

**Authors:** Christoph Linnemann, Undine E. Lang

**Affiliations:** University of Basel, Universitäre Psychiatrische Kliniken (UPK), Basel, Switzerland

**Keywords:** late-life depression, dementia, endocrine hypothesis, vascular hypothesis, neuroinflammation, amyloid hypothesis

## Abstract

Late-life depression is associated with significant cognitive impairment. Meta-analyses showed that depression is associated with an increased risk for Alzheimer’s disease (AD) and it might be an etiological factor for AD. Since late-life depression is often connected with cognitive impairment and dementia is usually associated with depressive symptoms, a simple diagnostic approach to distinguish between the disorders is challenging. Several overlapping pathophysiological substrates might explain the comorbidity of both syndromes. Firstly, a stress syndrome, i.e., elevated cortisol levels, has been observed in up to 70% of depressed patients and also in AD pathology. Stress conditions can cause hippocampal neuronal damage as well as cognitive impairment. Secondly, the development of a depression and dementia after the onset of vascular diseases, the profile of cerebrovascular risk factors in both disorders and the impairments depending on the location of cerebrovascular lesions, speak in favor of a vascular hypothesis as a common factor for both disorders. Thirdly, neuroinflammatory processes play a key role in the etiology of depression as well as in dementia. Increased activation of microglia, changes in Transforming-Growth-Factor beta1 (TGF-beta1) signaling, production of pro-inflammatory cytokines as well as reduction of anti-inflammatory molecules are examples of common pathways impaired in dementia and depression. Fourthly, the neurotrophin BDNF is highly expressed in the central nervous system, especially in the hippocampus, where it plays a key role in the proliferation, differentiation and the maintenance of neuronal integrity throughout lifespan. It has been associated not only with antidepressant properties but also a reduction of cognitive impairment and therefore could be involved also in AD. Another etiologic factor is amyloid accumulation, as plasma amyloid beta-42 independently predicts both late-onset depression and AD. Higher plasma amyloid beta-42 predicts the development of late onset depression and conversion to possible AD. However, clinical trials with antibodies against beta amyloid recently failed, i.e., Solanezumab, Aducanumab, and Crenezumab. An overproduction of amyloid-beta might simply reflect a form of synaptic plasticity to compensate for neuronal dysfunction in different kind of neurological and psychiatric diseases of multiple etiologies. The tau hypothesis, sex/gender specific differences, epigenetics and the gut microbiota-brain axis imply other potential common pathways connecting late-life depression and dementia. In conclusion, different potential pathophysiological links between dementia and depression highlight several specific synergistic and multifaceted treatment possibilities, depending on the individual risk profile of the patient.

## Introduction

Older adults with late-life depression often suffer from serious cognitive impairment without full recovery after successful antidepressant treatment. A relationship has been shown between history of depression and increased risk of dementia. Recent meta-analyses found that depression was associated with an increased risk for dementia and Alzheimer’s disease (AD).

Dementia is a clinical syndrome characterized by a progressive deterioration of cognitive function associated with impairment in activities of daily living (van der Flier and Scheltens, 2005) commencing mostly in late life. It is estimated that there will be approximately 4.6 million new cases every year worldwide, doubling every 20 years to 81.1 million by 2040 (Ferri et al., 2005). It is increasing in high-income countries and even more so in low- and middle-income countries (Kalaria et al., 2008). In late life the prevalence of depression is expected to rise and thus denotes new challenges for the mental health system. Mutual hypotheses to explain comorbidity suggest that depression might either be an early symptom of dementia, or a reaction to cognitive decline, or due to an overlap of both syndromes. Other hypotheses suggest that depression might increase susceptibility to dementia or act as a predictor, if not a causal factor for dementia (Lenoir et al., 2011). So far, no particular and distinctive symptom profile with substantial usefulness in the clinical setting has emerged as characteristic of late-life depression (Gallagher et al., 2010). More importantly, the intensity and the reporting of depressive symptoms in the elderly are suspected to be covert and not properly meeting the diagnostic criteria. Since late-life depression is often coupled with cognitive impairment and dementia may be associated with depressive symptoms, a simple differential diagnostic approach to distinguish between those syndromes is not always possible (Steffens and Potter, 2008). Depressive symptoms are reported in 30–50% of AD patients (Zubenko et al., 2003) and severe depressive episodes are reported in more than 10% of patients suffering from AD (Lopez et al., 2003) and in about 50% of patients with vascular dementia (Ballard et al., 2000; Park et al., 2007). The definitions of the terms “depression” and “dementia” are heterogeneous with blurred boundaries. Depression and dementia are not dichotomous symptoms or diagnoses. Depression can be considered as a symptom of dementia. Neuropsychiatric symptoms or behavioral and psychological symptoms are almost universal parts of dementia, as reviewed by van der Linde et al. (2016). The Cache County Study (Steinberg et al., 2008) revealed point and 5-year period prevalence of neuropsychiatric symptoms in dementia and found that participants most likely developed depression (77%), apathy (71%) and anxiety (62%). A 5-year longitudinal study of 223 patients with mild dementia and annual assessments in Western Norway (Vik-Mo et al., 2018) revealed that the most common symptoms were apathy (83%), depression (63%), appetite (63%), and aberrant motor behavior (60%). Connors et al. (2018) investigated the stability of neuropsychiatric subsyndromes in AD by principal component analyses and multiple-group confirmatory factor analyses. The findings suggest that the neuropsychiatric symptoms do not appear in distinctive subsyndromes that are stable over time. Neuropsychiatric symptoms in dementia have multiple overlapping relationships with one another. It is well-known that cognitive decline limits language skills as well as self-awareness of depressive symptoms. Depression and apathy due to dementia are difficult to differentiate. The problem of the correct diagnosis is foreshadowed by the large range of published prevalence rates for depression in AD from under 5% to nearly 50% (Weiner et al., 1994, 2002). Forgetfulness, difficulties in concentration, sleeping too much or not enough, reducing social contacts as well as loss of interest in hobbies mark additional areas of overlap for depression and dementia. Clinically, depression may masquerade as dementia, dementia may pose as depression, and dementia and depression may coexist. Neuropsychological tests and psychiatric exploration may not differentiate between reversible cognitive deficits due to depression and persistent cognitive impairment due to dementia. Widely used rating scales – in studies as well as in clinical daily routine – assessing the severity of depression are not always helpful for demented patients. The Montgomery–Asberg Depression Rating Scale – MADRS (Montgomery and Asberg, 1979) is not validated for demented patients (Holroyd and Clayton, 2000; Conn and Thorpe, 2007). The Hamilton Depression Rating Scale – HAM-D (Hamilton, 1960) has not been validated in severely demented patients (Lichtenberg et al., 1992). Interestingly, antidepressant drugs have proven efficacy in non-demented populations (Cipriani et al., 2018), but revealed negative outcomes in randomized placebo-controlled trials with antidepressants in dementia (Leong, 2014; Leyhe et al., 2017): venlafaxine (75 mg daily) for 6 weeks, assessed by MADRS in 31 patients (de Vasconcelos Cunha et al., 2007); fluoxetine (maximum 40 mg daily) for 6 weeks, assessed by HAM-D in 41 patients (Petracca et al., 2001) and imipramine (83 mg daily) for 8 weeks, assessed by HAM-D in 61 patients (Reifler et al., 1989). Pharmacological interventions based on serotonergic and noradrenergic etiology were mostly disappointing. A promising overlap between dementia and depression might be the glutamatergic signaling, namely the dysfunction of *N*-methyl-D-aspartate (NMDA) receptor complex signaling. NMDA receptor antagonists feature antidementia and antidepressant potential [for review see Khundakar and Thomas (2015)]. In a review by Butters et al. (2008), the hypothesis has been postulated that depression leads to subsequent cognitive impairment and dementia.

## The Endocrine Hypothesis

One of the most important endocrine components to respond to stress is the secretion of corticosteroid hormones. The neuroendocrinology of depressive patients – as far as the hypothalamic-pituitary adrenal (HPA) axis is concerned – shares common characteristics with that of rats that are chronically stressed (Checkley, 1996). Moreover, there is evidence for an increased central HPA axis activation in animal models of chronic stress (Herman et al., 1989; Angulo et al., 1991; de Goeij et al., 1992; Bartanusz et al., 1993; Harbuz et al., 1993) as well as in human depression (Ur et al., 1992; Raadsheer et al., 1994; Young et al., 1994). Secondly, the impaired negative feedback can be observed in animal models of chronic stress (Sapolsky et al., 1984; Young et al., 1990) as well as in depression (Carroll et al., 1981; Young et al., 1991). Thirdly, the hypertrophy of the adrenal gland can be found in chronically stressed animals (Herman et al., 1995) as well as in depressive patients (Dorovini-Zis and Zis, 1987; Nemeroff et al., 1992; Rubin et al., 1995). In line with these observations, the dexamethasone suppression test can be striking in depressive patients (Arana and Mossman, 1988). The hippocampus contains corticosteroid receptors (McEwen, 1999). Stress conditions as well as an exogenous application of glucocorticoids can cause hippocampal neuronal damage as well as cognitive impairment (Levy et al., 1994; Cereseto et al., 2006). Stress exposure causes volume reductions of the hippocampus, impairs dendritic complexity of neurons in the CA3 and affects neurogenesis in the dentate gyrus (Gould et al., 2000; Czeh and Lucassen, 2007). Increased levels of cortisol serum are associated with AD biomarkers in CSF and serum cortisol and CSF tau levels are negatively correlated (Laske et al., 2009). Similarly, hyperactivation of the hypothalamic-pituitary-adrenal (HPA) axis and increased levels of cortisol are most consistently observed findings in up to 70% of depressed patients (Porcelli et al., 2011). Impaired hippocampal plasticity may be related to cognitive impairment due to depression. A large number of MRI studies investigated the hippocampus subfield volumetrics in depressive patients compared to controls: As summarized by Maller et al. (2018), the findings were partly inconsistent. No significant differences concerning various hippocampal subfields in depressive patients compared to controls or correlations with illness duration or number of episodes have been found (Cho et al., 2010; Cole et al., 2010; Huang et al., 2013; Lindqvist et al., 2014; Na et al., 2014; Travis et al., 2015; Treadway et al., 2015; Han et al., 2016; Maller et al., 2018). Hippocampal tail volume was discussed as being a biomarker for sensitivity to treatment with antidepressant medication (Maller et al., 2018). The association of hippocampal volume and dementia, especially AD, is well known and has been refined several times (Allison et al., 2019).

## The Vascular Hypothesis

Clinically, the onset or worsening of a depression after the onset of vascular disease, the profile of cerebrovascular risk factors in depressive patients, and the impairments depending on the location and extent of a cerebrovascular lesion, as well as a poor response to antidepressant medication speak in favor of a vascular depression hypothesis (Alexopoulos, 2019). Several neuroimaging findings also support this hypothesis, namely – amongst others – low blood flow in the precuneus, cuneus, in fronto-cingulate-striatal areas as well as temporal, occipital, and parietal lobes, a resting functional connectivity pattern as postulated in depression and changes suggesting limbic hyperactivation. Circulating markers of endothelial dysfunction and flow mediated vascular dilatation also support the vascular depression hypothesis. However, the change of depressive symptoms over time, and the fact that infarcts were not associated with the severity of depression, cannot be readily brought in line with this hypothesis (Alexopoulos, 2019). Moreover, patients suffering from ischemic lesions do not necessarily develop a depression. Epidemiological investigations focus on the comorbidity of depression and dementia with cardiovascular diseases, i.e., heart failure, however, the prognostic role of depression and the impact of heart failure as a risk factor for dementia needs further investigation (Adelborg, 2018).

## The Neuroinflammation Hypothesis

Neuroinflammatory processes play an important role in the etiology of depression (Bhattacharya et al., 2016) as well as dementia (Knezevic and Mizrahi, 2018). Increased activation of microglia could be detected in depression (Setiawan et al., 2015) and in AD (Knezevic and Mizrahi, 2018). The anti-inflammatory cytokine Tumor Necrosis Factor-beta1 (TGF-beta1) is important for memory formation and synaptic plasticity and is reduced in depression in correlation with depression severity. A deficit in TGF-beta1 signaling pathway is common in depression and AD (Caraci et al., 2018). In a nutshell, the inflammation hypothesis of late life depression is underpinned by typical old-age immune responses [for summary see Alexopoulos (2019)] like production of pro-inflammatory cytokines as well as reduction of anti-inflammatory molecules, the insufficient clearance of neurotoxic molecules, neuronal loss and reduced neurogenesis. Cytokines lead to (1) the induction of enzymes that reduce the production of serotonin, (2) dysregulation of the glutamate system, (3) excitotoxicity and a reduced production of neurotrophic factors that are important for neuroplasticity and neurogenesis, and (4) oxidative stress, affecting glial cells in the prefrontal cortex and the amygdala. Inflammation impairs the function of glucocorticoid receptors. The increase of inflammatory markers is associated with depression severity as well as with cognitive impairment in depression. A treatment with antidepressant medication reduces inflammation markers, as well as non-steroidal anti-inflammatory drugs might have antidepressant effects in depressive persons.

The role of the gut microbiota-brain axis in affective disorders is subject of promising interventions with pro- and prebiotics as well as fecal microbiota transplants [as reviewed by Carlessi et al. (2019) and Peirce and Alvina (2019)]. The gut microbiota-brain axis could be a common pathway in late life depression and dementia. The recent review by Panza et al. (2019c) provided evidence for a gut microbial hypothesis in dementia with the appeal to test antibacterial therapy in AD.

## The Neurotrophin Hypothesis

The neurotrophin BDNF is highly expressed in the central nervous system, particularly in the hippocampus where it plays a key role in the proliferation, differentiation and the maintenance of neuronal integrity throughout lifespan (Lipsky and Marini, 2007). In AD, there is an association between the rate of cognitive decline and BDNF serum levels, amyloid beta (Aβ1-42) plasma levels and the degree of platelet activation (activated GP IIb-IIIa and P-selectin) (Laske et al., 2010; Stellos et al., 2010; Laske et al., 2011). Furthermore, up-regulation of serum BDNF after pharmacotherapy in AD patients revealed a reduction of cognitive impairment and therefore could mirror a neuroprotective effect (Leyhe et al., 2008, 2009). Synaptic plasticity in neuronal networks playing a role in depression is regulated by BDNF (Schinder and Poo, 2000; Pittenger and Duman, 2008). Moreover, stress-induced deficits in structural and synaptic plasticity may be reversed by up-regulation of BDNF, enhancing cognitive flexibility and resilience against depression. Depressed and demented patients show reduced BDNF levels which are increased by antidepressant treatment (Karege et al., 2002; Shimizu et al., 2003; Aydemir et al., 2005; Gervasoni et al., 2005; Lee et al., 2006, 2011; Kim et al., 2007; Brunoni et al., 2008; Sen et al., 2008; Deuschle et al., 2013; Ricken et al., 2013). BDNF polymorphism and serum level are related to depression, anxiety, neuroticism and serotonergic neurotransmission (Hellweg et al., 2002, 2008; Lang et al., 2002, 2004, 2005, 2006, 2007, 2009). An augmentation with lithium is an evidence-based antidepressant therapy and leads to increasing BDNF levels (Ricken et al., 2013). Of note, a common feature among depressed patients is insomnia (Steiger and Kimura, 2010). Giese et al. (2014) present data on the relation between BDNF levels and sleep disturbances, whereas patients suffering from insomnia revealed reduced BDNF levels.

## The Senescence Hypothesis

Various stimuli cause cellular senescence and a senescence-associated secretory phenotype, i.e., short/dysfunctional telomeres, non-telomeric DNA damage, oncogenes/oncogenic mutations, mitogenic/stress signals, overexpressed cell cycle inhibitors, and chromatin instability (Coppe et al., 2010). Senescence is linked to depression and could be a future therapeutic target (Diniz, 2018).

The role of telomere length and shortening as an indicator of cellular aging is discussed as a mechanism for stress-related depression (Boccardi and Boccardi, 2019) as well as for Alzheimer’s dementia (Boccardi et al., 2015; Nudelman et al., 2019).

Another link for the conversion of affective disorders to dementia is glycogen synthase kinase 3 (GSK-3) that might be an etiological factor for depression and dementia (Terao et al., 2019). Lithium inhibits GSK-3 and is effective for affective disorders and cognitive impairment (Terao et al., 2006; Nunes et al., 2007; Kessing et al., 2008; Gerhard et al., 2015).

## The Amyloid Hypothesis

According to Mahgoub and Alexopoulos (2016), amyloid accumulation is related to depression by means of frontolimbic impairment. Higher plasma amyloid beta-42 predicts the development of late-life depression and conversion to AD (Blasko et al., 2010). Increased levels of amyloid beta peptides could be associated with an amyloid-related depression (Morgese et al., 2015, 2017; Schiavone et al., 2017). In elderly persons without cognitive deficits, increased amyloid burden was related to depression (Yasuno et al., 2016; Donovan et al., 2018). The amyloid hypothesis is extensively discussed as primary cause for synaptic dysfunction and neurodegeneration in AD (Hardy and Higgins, 1992; Hardy and Selkoe, 2002). There is a huge amount of data that supports the hypothesis that amyloid beta related toxicity plays a role in AD [summarized in Herrup (2015)]: For example, APOE variants are a known risk factor for AD and demonstrated an effect on amyloid beta clearance; overexpression of human APP in mice results in the formation of plaques; transgenic mice for human APP demonstrate memory impairment; amyloid beta shows toxicity for cultured neurons; human APP overexpressed in fruit flies leads to neurodegeneration; amyloid plaques can be detected more frequent in AD brains; presence of plaques is related to greater risk for developing AD. There is, however, also a large amount of recent data underpinning that the claim for the absolute truth of this hypothesis as the primary cause for AD cannot be maintained, as summarized by Herrup (2015). Most importantly, several clinical trials with antibodies against beta amyloid based on this hypothesis failed. Solanezumab was not successful in phase 3 clinical trials (McCartney, 2015). Phase 3 clinical studies with Aducanumab were canceled in March 2019 because an interim analysis revealed that the trials were unlikely to meet the primary endpoint. However, after a reanalysis in October 2019, Biogen announced that the company intends to seek regulatory approval (Biogen, 2019). Concerning Crenezumab, Roche has recently announced discontinuation of the Phase III studies after a pre-planned interim analysis (Hoffmann-La Roche, 2019).

Amyloid beta has an important physiological role for the brain function, e.g., for neurogenesis, synaptic plasticity, memory and neuronal survival; and an overproduction of amyloid-beta might simply reflect a form of synaptic plasticity to compensate for neuronal dysfunction in different kind of neurological and psychiatric diseases of multiple etiologies, including cognitive (e.g., AD) as well as affective disorders, as reviewed by Panza et al. (2019b). This hypothesis of an amyloid beta overexpression as a compensatory attempt in terms of a repair mechanism is in line with observations that anti-amyloid beta drugs, e.g., beta-site amyloid precursor protein-cleaving enzyme 1 (BACE-1) inhibitors, may induce or worsen psychiatric disturbances in cognitively impaired patients (Egan et al., 2019a, b; Henley et al., 2019; Panza et al., 2019a).

## The Tau Hypothesis

Rapp et al. (2006, 2008) investigated the interaction between depression and neurofibrillary tangles in AD patients and indeed found increased neurofibrillary tangles in AD patients with a comorbid depression. However, Tsopelas et al. (2011) found that a history of late-life depression was not associated with neurofibrillary tangles in brains whose donors had no history of dementia. Longitudinal clinical-pathologic cohort studies with almost 2000 participants do not support the tau hypothesis that depression is associated with neurofibrillary tangles (Wilson et al., 2016).

## Gender Differences

Gender specific associations and differences in resilience to stress (Hodes and Epperson, 2019) and in depression and dementia are widely discussed, with partly divergent results (Fuhrer et al., 2003; Kessing and Nilsson, 2003; Dal Forno et al., 2005; Simons et al., 2006; Artero et al., 2008; Chen et al., 2008; Noale et al., 2013; Mirza et al., 2014; Kim et al., 2015; Heser et al., 2020). A female to male prevalence ratio of 2:1 is described for depression (Bromet et al., 2011) and dementia (Ferretti et al., 2018). The gender differences in published prevalence rates of depression might be partly explained by the fact that men are less willing to seek psychiatric help (Kessler et al., 1981; Ferretti et al., 2018), leading to a possible underestimation of depression in men. The sex differences in life expectancy might also have an influence on the ratio for dementia. Nevertheless, sex-specific biological features, e.g., the effect of estrogens on mood and/or cognition (Kawas et al., 1997; Waring et al., 1999; Wang et al., 2000), might modulate the risk for affective and cognitive disorders. Dal Forno et al. (2005) and Heser et al. (2020) provided evidence that the association between depression and dementia is stronger in the male population.

## Epigenetics

Several reviews exist about the role of epigenetics (meaning “on top of” genetics and without changes of DNA sequence), in the pathogenesis of depression (e.g., Menke and Binder, 2014; Nestler, 2014; Saavedra et al., 2016; Lin and Tsai, 2019) and dementia (e.g., Maloney and Lahiri, 2016; Fenoglio et al., 2018; Lemche, 2018; Stoccoro and Coppede, 2018; Sujeetha et al., 2018). To be honest, epigenetic results are fairly variable for depression and dementia, and the lack of common stable epigenetic patterns makes it difficult to relate reliable epigenetic factors in depression with the risk of AD, as summarized by Herbert and Lucassen (2016). Further limitations addressing epigenetic research with correlational studies are discussed in the following section.

## Limitations

When assessing correlational studies, it is always important to keep in mind the advantages and limitations of this approach (Asamoah, 2014): A correlation does not necessarily imply causation. Moreover, the dilemma of directionality (the “chicken-and-egg problem”) cannot be sufficiently addressed by a correlational approach: It cannot be concluded that changes in variable A might cause changes in variable B, but that also changes in variable B might cause changes in variable A. With respect to a multifactorial complexity of affective symptoms and cognitive impairment in humans, the variables A and B are supposed to be related to variables C, D, E, ….

On the other hand, causation does indeed imply a correlation. This makes it an important tool for the falsification of a hypothesis. Bearing this consideration in mind, the results of correlational studies can offer interesting ideas for further research or improved diagnostic and/or therapeutic steps. Correlational studies are extremely helpful to formulate an interesting hypothesis for experimental research. However, experimental approaches in the field of pathogenesis of depression and dementia in humans are challenging or impossible because important variables cannot be manipulated or their manipulation would be unethical. Moreover, an experimental approach using animal models for affective and cognitive diseases is limited and cannot cover all aspects of human pathology. Another challenge is the use of a representative sample and the generalization to the population of patients with depression and/or dementia.

## Conclusion

The pathogenesis of both syndromes themselves is not understood so far – making it more difficult to describe common pathways. There is a vast amount of literature showing several potential links between dementia and depression highlighting the multifactorial complexity of both syndromes. In accordance with these multifaceted pathologies different individualized strategies (antiinflammatory, psychotherapeutic, antidepressant, antihypertensive, and endocrinological) might help to overcome the pathophysiology of dementia and depression in an individualized treatment regimen depending on the individual risk factors. However, a common drawback of all links between affective and cognitive impairments mentioned and discussed above is the lack of specificity for depression and/or dementia. When searching the literature for links between these fields, many interesting intersections can be found – but also many limitations and downsides that do not the support the respective hypotheses. To be honest, all pathways connecting dementia and late life depression that are found so far can only explain a small part of the story and have strong limitations. Correlational approaches have severe limitations, as a correlation does not necessarily imply causation. The dilemma of directionality (the “chicken-and-egg problem”) as well as the third-variable problem cannot be solved. Experimental research in humans is impeded by ethical limits or the fact that certain variables cannot be manipulated. Moreover, animal models cannot address all relevant aspects of the human pathology in depression and dementia. The review of the literature shows that most publications in this field end with an outlook and perspective for prevention and/or therapy of cognitive or affective disorders that are somehow contrived and artificial hypotheses – far away from a quantum jump and breakthrough for applied therapies – due to the multifactorial complexity of both syndromes.

## Author Contributions

CL and UL performed the literature research and wrote the manuscript.

## Conflict of Interest

The authors declare that the research was conducted in the absence of any commercial or financial relationships that could be construed as a potential conflict of interest.
